# Risks of accidents involving biological material in Primary Care: analysis of the state of São Paulo (2020-2024)

**DOI:** 10.47626/1679-4435-2025-1480

**Published:** 2025-09-25

**Authors:** Danilo Panebianco, Gabriel Demarchi, Gabryel Roberto da Costa, Lorena Passos Viana, Marcio Amaro Sena Curvello, Ricardo Toshio Enohi

**Affiliations:** 1 Curso de Medicina, Universidade de Ribeirão Preto, Guarujá, SP, Brazil

**Keywords:** occupational accident, risk factors, biomaterial, primary health care., acidente de trabalho, fatores de risco, material biológico, atenção primária à, saúde.

## Abstract

**Introduction:**

Work accidents due to exposure to biomaterials represent a common risk in the routine of primary health care professionals, due to insufficient training and partial adherence to biosafety standards.

**Objectives:**

To identify, between 2020 and 2024, the risk factors associated with work accidents involving exposure to biological material in Primary Health Care and Family Health Strategy professionals in the state of São Paulo.

**Methods:**

Ecological study based on data from the Notifiable Diseases Information System database, including Primary Health Care professionals. Statistical analysis was performed using the Mann-Whitney and Kruskal-Wallis tests.

**Results:**

A total of 289 cases were reported, with white women between 35 and 49 years old predominating. Community health agents and nursing technicians had the highest number of notifications. Dental procedures were the main circumstance of accidents. Most were vaccinated against hepatitis B and tested negative for hepatitis B surface antigen, antibodies to hepatitis B surface antigen, hepatitis C virus, and human immunodeficiency virus.

**Conclusions:**

Work accidents due to exposure to biomaterial have a defined epidemiological profile, which facilitates the implementation of targeted measures, especially for the most vulnerable group - community health agents - aimed at improving professional training, reinforcing safe practices, and strengthening preventive strategies.

## Abstract (pt)

**Introdução:**

Os acidentes de trabalho por exposição a material biológico representam um risco comum na rotina dos profissionais da atenção primária, devido à falta de capacitação e adesão parcial às normas de biossegurança.

**Objetivos:**

Identificar, entre os anos de 2020 e 2024, os fatores de risco associados aos acidentes de trabalho por exposição a material biológico nos profissionais da Atenção Primária à Saúde e Estratégia de Saúde da Família no estado de São Paulo.

**Métodos:**

Estudo ecológico coletado na base de dados do Sistema de Informação de Agravos de Notificação que incluiu profissionais de saúde da Atenção Primária. A análise estatística foi realizada pelos testes Mann-Whitney e Kruskal-Wallis.

**Resultados:**

Foram reportados 289 casos, predominando mulheres brancas entre 35 e 49 anos de idade. Os agentes comunitários de saúde foram os mais notificados. Procedimentos odontológicos foram a principal circunstância de acidente. A maioria é vacinada para hepatite B e testou negativo para as sorologias de antígeno de superfície da hepatite B, anticorpo contra o antígeno de superfície da hepatite B, anticorpo contra o vírus da hepatite C e anticorpo contra o vírus da imunodeficiência humana.

**Conclusões:**

Os acidentes de trabalho por exposição a material biológico apresentam um perfil epidemiológico definido, o que facilita o direcionamento de medidas focadas sobretudo no grupo mais vulnerável - os agentes comunitários de saúde - visando à melhora da capacitação profissional, reforço de práticas seguras e estratégias preventivas.

## INTRODUCTION

An occupational accident is defined as any event occurring during the performance of professional activities, whether in a corporate environment or in domestic services, that results in physical injury or functional impairment of the worker.^1,2^ Such events may lead not only to fatalities but also to partial or total reduction of work capacity, either temporary or permanent, directly affecting health and functional performance.^3^ In terms of classification, occupational accidents are grouped into 3 main categories: commuting accidents, which occur during travel between home and the workplace or vice versa; typical accidents or occupational diseases, triggered or produced by work activity; and work-related diseases, which result from specific conditions under which the work is performed, being directly related to the occupational environment and its demands.^4,5^

Among the different types of occupational accidents, those caused by exposure to biological material (E-BMAs) occur when a worker comes into direct or indirect contact with contaminated substances (e.g., blood, blood-contaminated fluid, serum, and plasma) through sharps injuries or splashes on mucous membranes and nonintact skin. Consequently, the risk of infection increases, as the worker becomes susceptible to more than 60 different species of microorganisms, including viruses - particularly hepatitis B virus (HBV), hepatitis C virus (HCV), and HIV - as well as bacteria, fungi, and protozoa.^2,6^

Mandatory reporting of E-BMAs has been in place in Brazil since 2004, under Ordinance No. 104 of January 25, 2011, through the Notifiable Diseases Information System (Sistema de Informação de Agravos de Notificação, SINAN).^6,7^ Professionals who most frequently use this reporting system are those working directly or indirectly in health care, who represent the groups most exposed to biological agents: physicians, nurses, and nursing assistants.^8,9^ This is explained by their direct interaction with patients, long working hours, overload, and physical and emotional strain. Risk is further aggravated by factors such as the large workforce, insufficient training, inadequate use of materials and personal protective equipment (PPE), overconfidence, inattention, and failure to adopt standard precautions.^8,10^

According to the Occupational Health and Safety Observatory, Brazil recorded approximately 612,900 occupational accidents in 2022. The health sector accounted for a significant share, representing 10% of the cases reported to the National Institute of Social Security (Instituto Nacional do Seguro Social, INSS).^2^ Between 2010 and 2016, 243,621 cases were reported among health professionals, with an annual average of 34,803 notifications. The incidence rate ranged from 3.65 to 24.7 cases per 1,000 professionals per year, depending on the state. Most victims were young women. In 2016, 76.4% of accidents involved members of the nursing staff.^11^ The high number of reports is related not only to the increase in the accidents themselves but also to improvements in SINAN and to the larger number of active health professionals, especially physicians and nursing technicians.^7^

Although the debate has focused mainly on hospital-based care practices, it is important to recognize that such events also occur frequently among primary health care (PHC) and Family Health Strategy professionals.^9,12^ In the daily practice of PHC units, physicians, nurses, nursing technicians, and nursing assistants perform small and intermediate outpatient procedures, minor urgent procedures, and advanced life support. These include injectable drug administration, laboratory specimen collection, minor sutures, and wound dressing - all of which are associated with biological risk.^13^

Because of the evident scarcity of E-BMA records and reports in PHC,^12^ it is essential to understand the profile of affected workers, since this allows anticipation of the main risk factors and improvement of preventive strategies targeted to the most vulnerable groups. The present study therefore aimed to identify the risk factors associated with E-BMAs in the state of São Paulo, involving PHC and Family Health Strategy professionals between 2020 and 2024.

## METHODS

This was an ecological study based on secondary data extracted directly from SINAN and accessed through the Tabnet platform, available on the website of the Department of Informatics of the Brazilian Unified Health System (Departamento de Informática do Sistema Único de Saúde, DATASUS). Data analyzed referred to the state of São Paulo, covering the period of 2020-2024. The analysis of accidents involving E-BMAs focused on health professionals working in PHC and the Family Health Strategy.

Data collected included PHC workers of different ethnicities, aged 15-79 years, with varying educational backgrounds (elementary, secondary, or higher education, either incomplete or complete).

Occupations selected from SINAN included physicians and nurses as well as nursing technicians and assistants, community health workers (CHWs), dentists, and oral health technicians.

The study also evaluated the use of PPE (e.g., mask, gloves, goggles, gown, boots, and face shield) at the time of exposure; the type of organic material involved in the accidents (blood, blood-contaminated fluid, serum and plasma, amniotic fluid, and others); and the circumstances of exposure (administration of intravenous, intradermal, intramuscular, and subcutaneous medications; surgical, laboratory, and dental procedures; material washing; handling of sharps containers; capillary blood glucose testing; venipuncture; epithelial nodule puncture; needle recapping; and improper disposal of biological material in trash bins or on the floor). In addition, the study assessed the agents responsible for accidents (needles with and without lumen, intracath, blades/lancets, and other sharp instruments) and the type of exposure caused by these agents (percutaneous, mucosal, intact skin, and nonintact skin).

Vaccination status against hepatitis B and serological results for hepatitis B surface antigen (HBsAg), antibody to hepatitis B surface antigen (anti-HBs), anti-HCV, and anti-HIV were also evaluated. Case outcomes were classified as lost to follow-up, unknown/blank, discharge with negative source patient, and discharge with or without seroconversion. Finally, the issuance of work accident reports (*comunicação de acidente de trabalho*, CAT) was recorded.

After collection, data were organized in spreadsheets and subsequently processed using Jamovi software, version 2.3.28, for statistical analysis and synthesis of the information collected.

Statistical analysis was performed using nonparametric tests, with a significance level of 5% (p < 0.05). Associations between variables were assessed using the Mann-Whitney and Kruskal-Wallis tests.

## RESULTS

**Figure 1:**
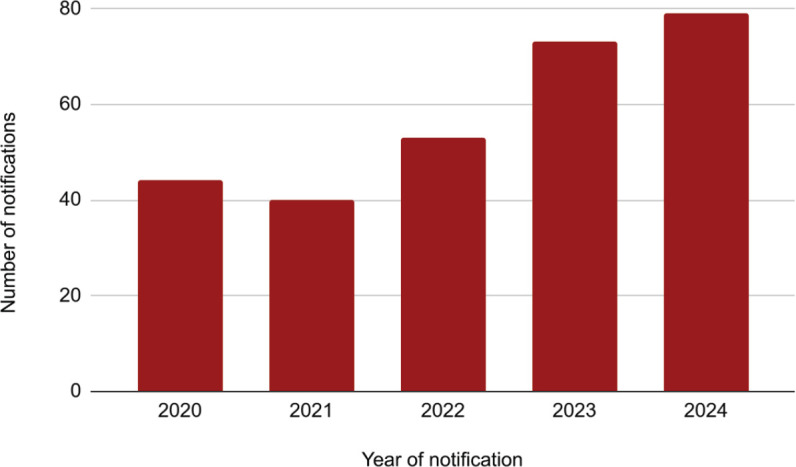
Number of reported occupational accidents involving exposure to biological material from 2020 to 2024 in the state of São Paulo.

Data collection yielded a total of 289 cases of E-BMAs. Figure 1 shows the years of notification, from 2020 to 2024, with 2024 recording the highest number of cases (27.34%) and 2021 the lowest (13.84%). A comparison between these 2 years shows that the number of notifications in 2024 nearly doubled compared with 2021.

As shown in Table 1, there was a significant difference in notifications between sexes (p = 0.012), with females being predominant (86.16%). Variables related to race, educational level, and age group also showed statistically significant differences, with p values < 0.001. Most individuals were White (65.05%) and aged 35-49 years (47.75%), followed by those aged 20-34 years (40.48%). Regarding education, most had completed secondary education (44.29%) or higher education (42.21%). The distribution of notifications by health occupation showed that CHWs (26.64%) and nursing technicians (22.49%) were the groups with the highest frequency of notifications, whereas dentists accounted for 11.42% of cases. The p value of 0.014 confirmed statistically significant differences in the distribution of notifications across occupations, suggesting that profession influences the occurrence or reporting of these events.

**Table 1:** Sociodemographic data of health professionals affected by occupational accidents involving biological material, 2020-2024

Variable	Absolute frequency	Relative frequency (%)
Sex		
Male	40	16.06
Female	249	86.16
Race		
Asian	6	2.08
White	188	65.05
Brown	76	26.30
Black	19	6.57
Education		
Completed elementary	2	0.69
Incomplete secondary	7	2.42
Completed secondary	128	44.29
Incomplete higher	30	10.38
Completed higher	122	42.21
Age group (years)		
15-19	1	0.35
20-34	117	40.48
35-49	138	47.75
50-64	31	10.73
65-79	2	0.69
Occupation		
Community health worker	77	26.64
Nursing assistant	28	9.69
Dentist	33	11.42
Nurse	26	9.00
Physician	40	13.84
Nursing technician	65	22.49
Oral health technician	20	6.92

Source: Information System for Notifiable Diseases (SINAN).

The characteristics of the accidents, presented in Table 2, showed that the most common type of exposure was percutaneous, with 222 reports (60.33%), followed by intact skin exposure, with 113 reports (30.71%). This difference was statistically significant (p = 0.001). Hollow-bore needles were identified as the main causative agent, accounting for 55.71% of all reports, with a significant variation among agents (p < 0.001). Blood was the predominant biological material involved, present alone in 89.27% of the cases, in addition to being mixed with other fluids in 5.54% of the reports, also showing a substantial difference (p < 0.001). Regarding the circumstances of the accidents, dental procedures (14.19%), administration of intramuscular medications (10.38%), and handling of sharps containers (9.34%) stood out, all of which also showed statistically significant differences (p < 0.001). Among the PPE used by professionals at the time of the accidents, the most frequent were masks (64.01%), gloves (60.21%), and gowns (47.40%), showing that more than 1 type of PPE was used in some cases.

**Table 2:** Data on accidents involving biological material and the use of personal protective equipment (PPE), 2020-2024

Variable	Absolute frequency	Relative frequency (%)
Type of exposure		
Mucosal exposure	18	4.89
Nonintact skin exposure	15	4.08
Intact skin exposure	113	30.71
Percutaneous exposure	222	60.33
Agent		
Hollow-bore needle	161	55.71
Solid needle	40	13.84
Intracath	1	0.35
Blade/lancet (any type)	46	15.92
Others	41	14.19
Organic material		
Blood-containing fluid	16	5.54
Amniotic fluid	2	0.69
Blood	258	89.27
Serum/plasma	2	0.69
Others	11	3.81
Circumstances of accident		
Intravenous drug administration	18	6.23
Intradermal drug administration	2	0.69
Intramuscular drug administration	30	10.38
Subcutaneous drug administration	12	4.15
Improper disposal on the floor	24	8.30
Improper disposal in the trash	13	4.50
Blood glucose testing	20	6.92
Instrument washing	10	3.46
Handling of sharps containers	27	9.34
Surgical procedure	17	5.88
Laboratory procedure	3	1.04
Dental procedure	41	14.19
Nodule puncture	3	1.04
Specimen collection puncture	19	6.57
Needle recapping	5	1.73
Other	45	15.57
PPEs		
Gown	137	47.40
Boots	23	7.96
Gloves	174	60.21
Mask	185	64.01
Face shield	37	12.80
Goggles	86	29.76

Source: Information System for Notifiable Diseases (SINAN).

According to Table 3, 283 individuals were vaccinated against hepatitis B, representing 97.92% of the accident victims. Regarding serological tests, most individuals tested negative for HBsAg (92.39%), anti-HBs (64.71%), and anti-HCV (94.12%), with p = 0.002 for all variables. The anti-HIV test had the highest number of negatives among all tests (96.19%), with p = 0.003. It is noteworthy that most individuals did not develop the anti-HBs antibody, despite having a complete vaccination schedule for hepatitis B. Moreover, among all tests not performed, the anti-HBs test showed the highest absence rate (25.61%).

**Table 3:** Clinical data of professionals affected by occupational accidents involving biological material and case outcomes, 2020-2024

Variable	Absolute frequency	Relative frequency (%)
Hepatitis B vaccination status		
Vaccinated	283	97.92
Not vaccinated	6	2.08
HBsAg		
Negative	267	92.39
Positive	1	0.35
Not performed	21	7.27
Anti-HBs		
Negative	187	64.71
Positive	28	9.69
Not performed	74	25.61
Anti-HCV		
Negative	1	0.35
Positive	272	94.12
Not performed	16	5.54
Anti-HIV		
Negative	1	0.35
Positive	278	96.19
Not performed	10	3.46
Case outcome		0.00
Abandonment	18	6.23
Discharge with seroconversion	1	0.35
Discharge - negative source patient	131	45.33
Discharge without seroconversion	76	26.30
Ignored/blank	63	21.80
Issuance of CAT		
Yes	194	67.13
No	37	12.80
Not applicable	8	2.77
Ignored/blank	50	17.30

Source: Information System for Notifiable Diseases (SINAN).

CAT = Work Accident Report; HBsAg = hepatitis B surface antigen; HCV = hepatitis C virus.

Of the total cases, 131 were discharged after being classified as negative source patients, followed by 76 discharged without seroconversion, indicating a favorable outcome. The p < 0.001 demonstrates that there was a statistically significant difference among the types of case outcomes.

Finally, Table 3 showed that most individuals (67.13%) issued a CAT. The p = 0.001 suggests that the issuance was not random but rather associated with the outcome and with characteristics that exposed workers to the risks of E-BMAs.

## DISCUSSION

In the present study, most E-BMAs involved female professionals (86.16%) and those of White ethnicity (65.05%). These data, with the exception of ethnicity, can be explained by the large female presence in health professions, which represent one of the categories most exposed to biological risks.^14^ The predominance of White individuals contrasts with previously published studies, which reported a higher prevalence of individuals of mixed ethnicity.^1,2^

Regarding age, the most affected group was between 35 and 49 years, as also observed in the study by Trindade et al.,^11^ which pointed out that one of the probable causes for this is overconfidence, stemming from years of experience, which leads workers not to adhere to standard precautions and the use of PPE.

Most professionals had completed high school (44.29%), followed by higher education (42.21%). This is possibly due to the predominant participation of nursing technicians and assistants in these activities. These findings are consistent with previous studies that identified a similar profile in different regions of Brazil.^6,10^

With regard to occupation, the results showed that among all the professions analyzed, CHWs had the highest incidence of accidents. Since 2017, the Brazilian National Primary Care Policy has included, among the functions of these professionals, procedures such as capillary blood glucose measurement and wound care, provided that they receive specific training on these techniques.^15^ According to the study by Galdino Júnior et al.,^16^ in addition to CHWs having a limited perception of biological risks in their daily practice, they also perform a variety of activities beyond the legal scope of their competencies, compounded by poor working conditions and gaps in professional training. The same study highlighted that the main activities that increase the risk of E-BMAs among these professionals include the improper disposal of sharps, finger pricking, changing diapers, and assisting with bathing during home visits.

After CHWs, nursing technicians represent the second professional category with the highest number of E-BMAs. This is due to their frequent performance of procedures involving the handling of sharps and biological fluids, with risks aggravated by their continuous contact with patients with infectious diseases.^11^

Blood was the most frequently involved biological material in the accidents (89.27%), similar to the study by Mangueira et al.^10^, which reported 81.54% of accidents with this type of material, and by Ornelas et al.^13^, with 84%.

The main route of exposure was percutaneous (60.33%), accounting for 80%-90% of infectious diseases among health professionals, as noted by Bertelli et al.^6^. The same study indicated that, after an accident, the risk of contracting HBV is 1 in 3; HCV, 1 in 30; and HIV, 1 in 300.

Hollow-bore needles were the main agents causing accidents (55.71%) due to their recurrent use in invasive practices, such as intramuscular drug administration (10.38%), a highly demanding and frequent activity in this context.^4^ Handling sharps containers (9.34%), when combined with manual handling, significantly increases the risk of accidental perforation.^17^

Regarding the use of PPE, most professionals were wearing masks (64.01%), gloves (60.21%), and gowns (47.40%) at the time of the accident, indicating partial adherence to biosafety standards. Among the factors that may explain this low adherence are work overload, lack of time, and issues related to insufficient specific training and limited institutional encouragement.^14^

Although the risk of contamination by pathogens such as HBV, HCV, and HIV is considered low, especially when biosafety and postexposure prophylaxis protocols are followed, serological monitoring of affected professionals remains essential to ensure worker safety.^7^ In this study, most of the injured professionals tested negative for HBsAg (92.39%), anti-HCV (94.12%), and anti-HIV (96.19%) during follow-up exams, indicating absence of active infection, immunization, or seroconversion.^10^ Despite the high adherence to hepatitis B vaccination (97.92%), anti-HBs also showed a large number of negative results (64.71%), leaving 187 workers susceptible to infection. This demonstrates that vaccination did not result in consolidated immunity against HBV, which may be related to incomplete vaccination schedules, vaccine failures, lack of post-vaccination testing, or individual factors affecting immune response.^18^ These findings align with the fact that, of all injured workers, 131 were classified as having a negative source, and 76 were discharged without seroconversion, demonstrating the effectiveness of postexposure measures and the importance of early diagnosis and proper follow-up.^13^

This study has some limitations regarding the use of secondary data, which may lead to underreporting of accidents, especially those considered less severe. Variations in data quality and completeness in reporting forms may also occur. Information on vaccination and PPE use is based on self-report or records, subject to bias. The lack of detailed data on the specific context of each accident further limits the analysis of contributing factors.

## CONCLUSIONS

This study revealed a predominant profile of E-BMA among female professionals, aged 35-49 years, of White ethnicity, and with a high school education. The most common form of exposure was percutaneous, with blood being the main biological material involved. Hollow-bore needles stood out as the most frequent causative agents. Regarding occupation, CHWs and nursing technicians were the most affected groups. In terms of vaccination coverage and clinical outcomes, most workers were vaccinated against hepatitis B, and most cases had favorable outcomes, with no seroconversion, as shown by the negative results for HBsAg, anti-HBs, anti-HCV, and anti-HIV in serological tests. It was also observed that, in most episodes, a CAT was issued, reflecting adequate case reporting.

The findings point to a defined epidemiological pattern, which can guide the adoption of health education measures aimed mainly at the most vulnerable group, CHWs, to improve technical training, adherence to standard precautions, and teamwork.
